# Supplementary material for: Steroids and Fatty Acid Esters from Cyperus sexangularis Leaf and Their Antioxidant, Anti-Inflammatory and Anti-Elastase Properties
Source: Molecules. 2023 Apr 13;28(8):3434. doi: 10.3390/molecules28083434 (PMC10141076; doi:10.3390/molecules28083434)
Supplement: Supplementary file 1 [file molecules-28-03434-s001.zip › molecules-2287851-supplementary.pdf]

Fig. S1

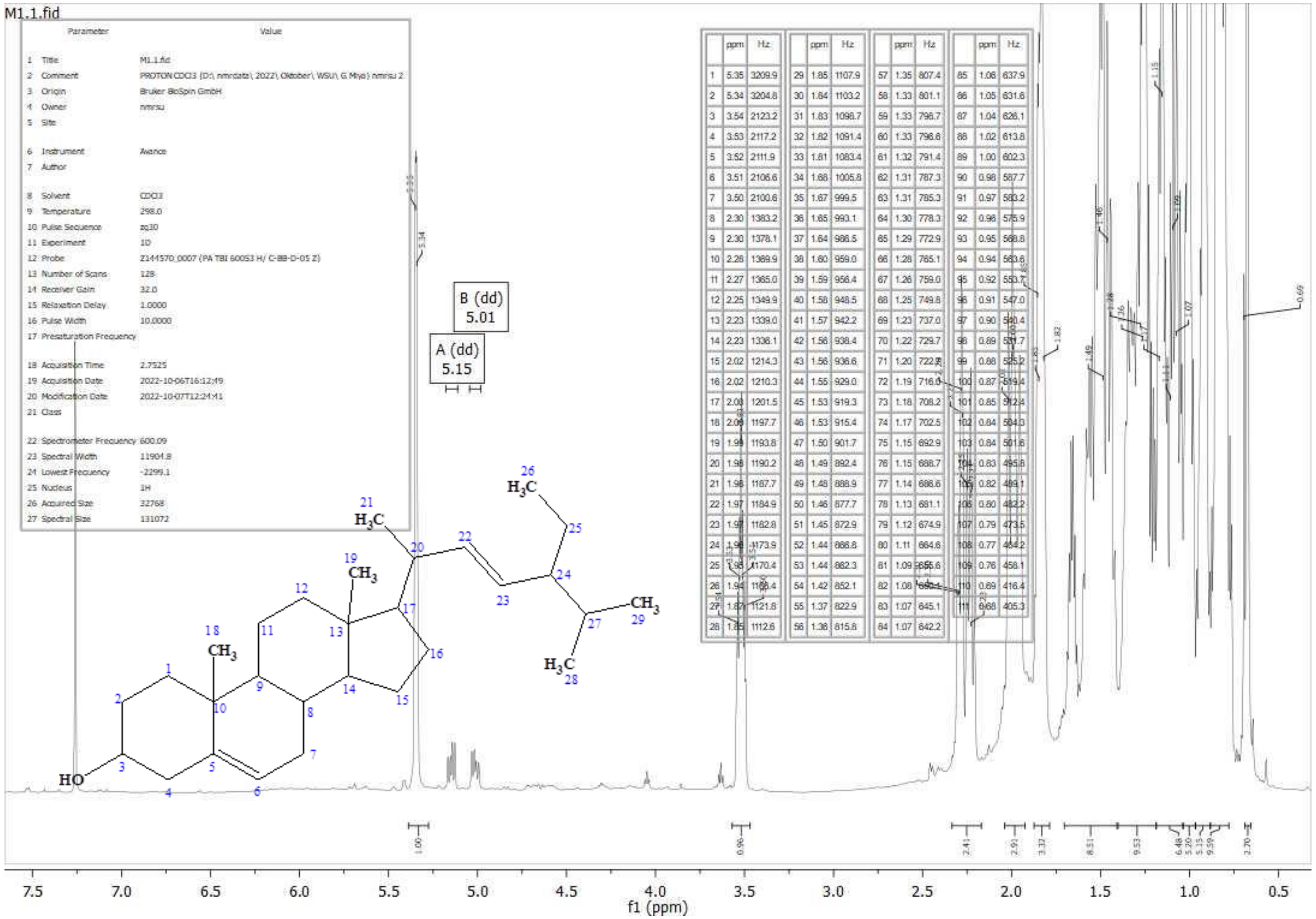

^{1}H NMR Spectrum of Compound 1 – Stigmasterol from the n-Hexane Leaf Extract of *Cyperus sexangularis* (CS)

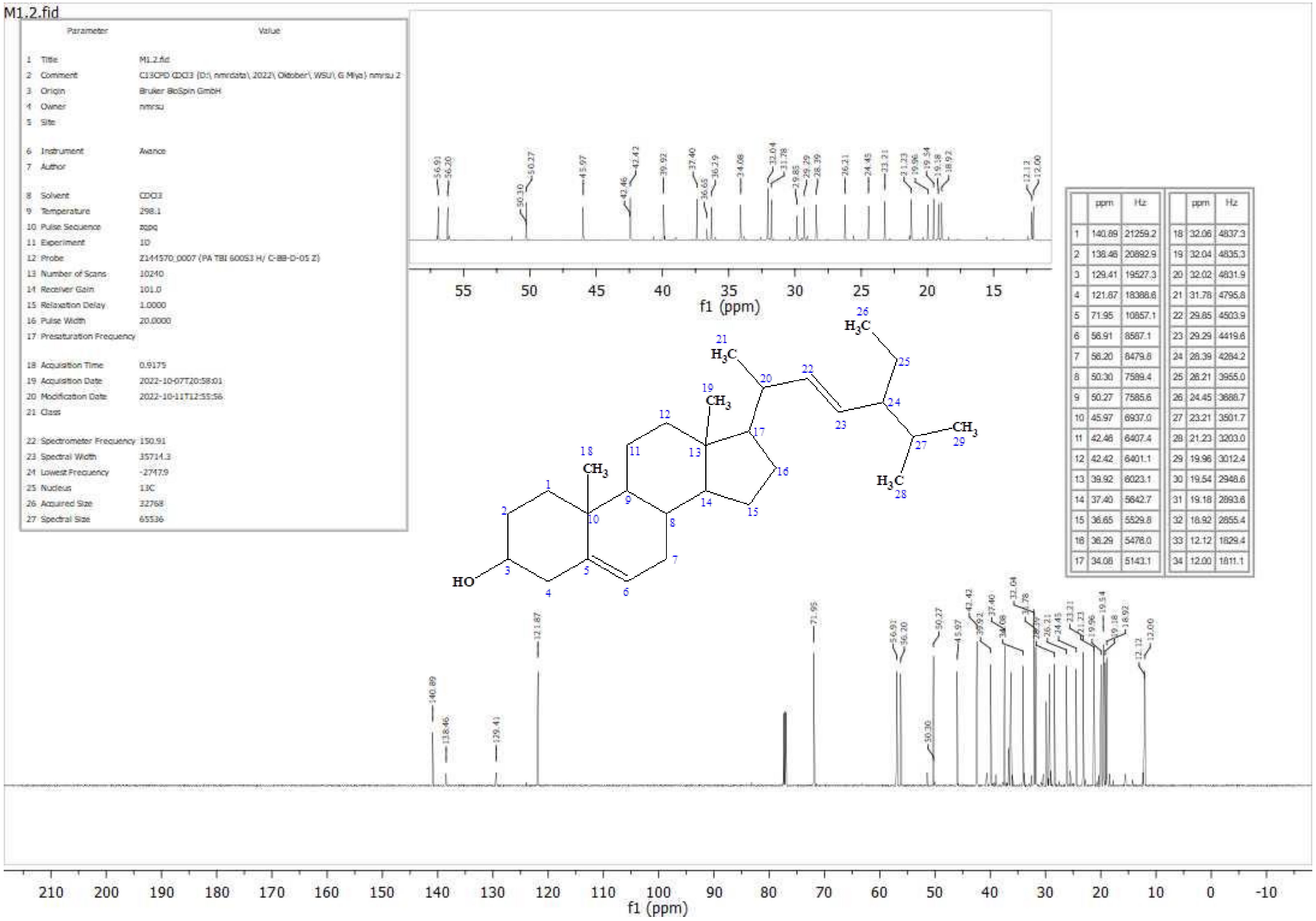

	Parameter	Value
1	Title	M1.2.fid
2	Comment	C13CPD CDCl3 {D:\ nmrdata\ 2022\ Oktober\ WSU\ G Miya} nmrsu 2
3	Origin	Bruker BioSpin GmbH
4	Owner	nmrsu
5	Site	
6	Instrument	Avance
7	Author	
8	Solvent	CDCl3
9	Temperature	298.1
10	Pulse Sequence	zgpg
11	Experiment	1D
12	Probe	Z144570_0007 (PA TBI 600S3 H/ C-BB-D-05 Z)
13	Number of Scans	10240
14	Receiver Gain	101.0
15	Relaxation Delay	1.0000
16	Pulse Width	20.0000
17	Presaturation Frequency	
18	Acquisition Time	0.9175
19	Acquisition Date	2022-10-07T20:58:01
20	Modification Date	2022-10-11T12:55:56
21	Class	
22	Spectrometer Frequency	150.91
23	Spectral Width	35714.3
24	Lowest Frequency	-2747.9
25	Nucleus	13C
26	Acquired Size	32768
27	Spectral Size	65536

	ppm	Hz		ppm	Hz
1	140.89	21259.2	18	32.06	4837.3
2	138.46	20892.9	19	32.04	4835.3
3	129.41	19527.3	20	32.02	4831.9
4	121.87	18388.6	21	31.78	4795.8
5	71.95	10857.1	22	29.85	4503.9
6	56.91	8587.1	23	29.29	4419.6
7	56.20	8479.8	24	28.39	4284.2
8	50.30	7589.4	25	26.21	3955.0
9	50.27	7585.6	26	24.45	3688.7
10	45.97	6937.0	27	23.21	3501.7
11	42.46	6407.4	28	21.23	3203.0
12	42.42	6401.1	29	19.96	3012.4
13	39.92	6023.1	30	19.54	2948.6
14	37.40	5642.7	31	19.18	2893.6
15	36.65	5529.8	32	18.92	2855.4
16	36.29	5476.0	33	12.12	1829.4
17	34.08	5143.1	34	12.00	1811.1

^{13}C NMR Spectrum of Compound 1 – Stigmasterol from the n-Hexane Leaf Extract of CS

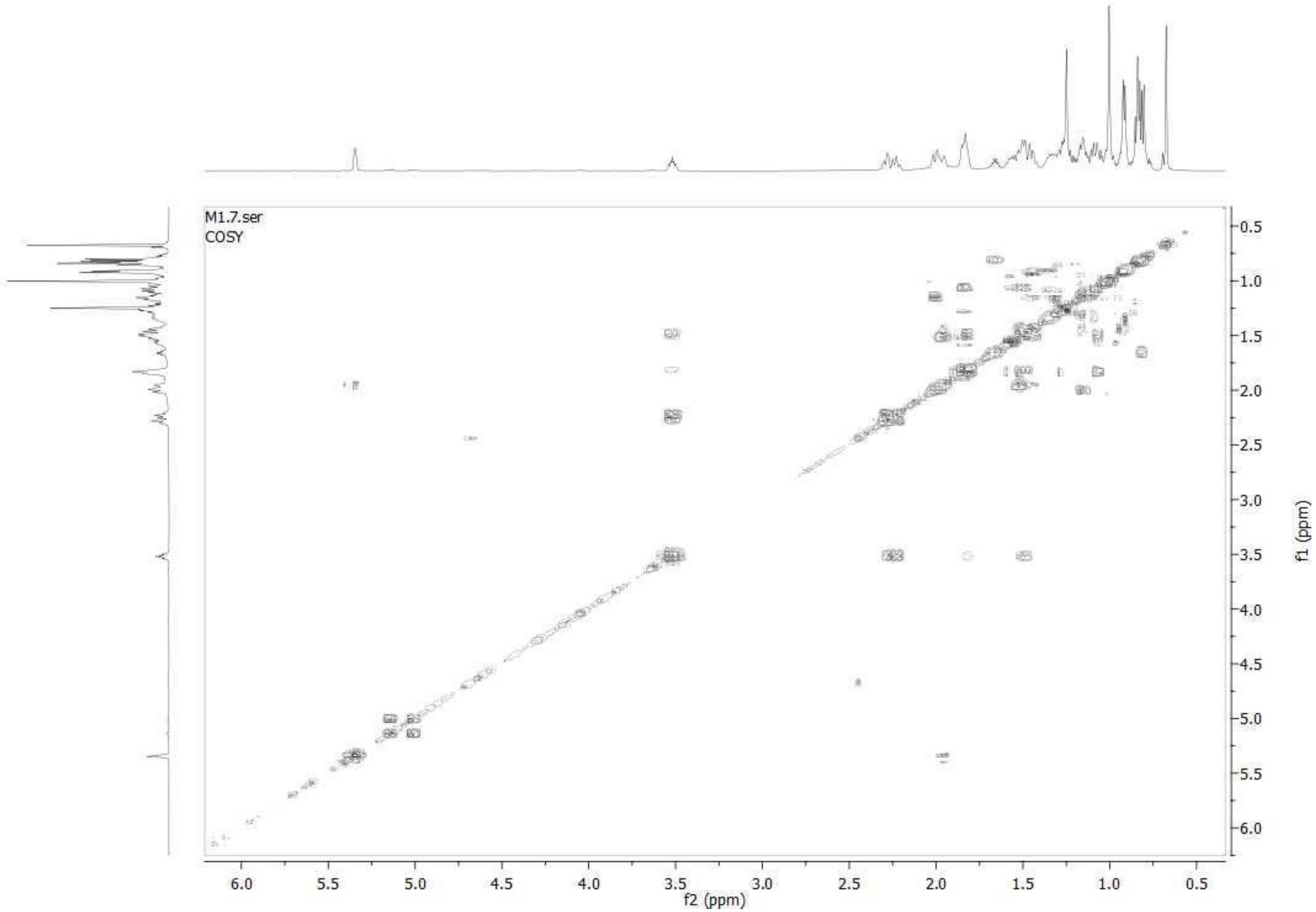

COSY NMR Spectrum of Compound 1 – Stigmasterol from the n-Hexane Leaf Extract of CS

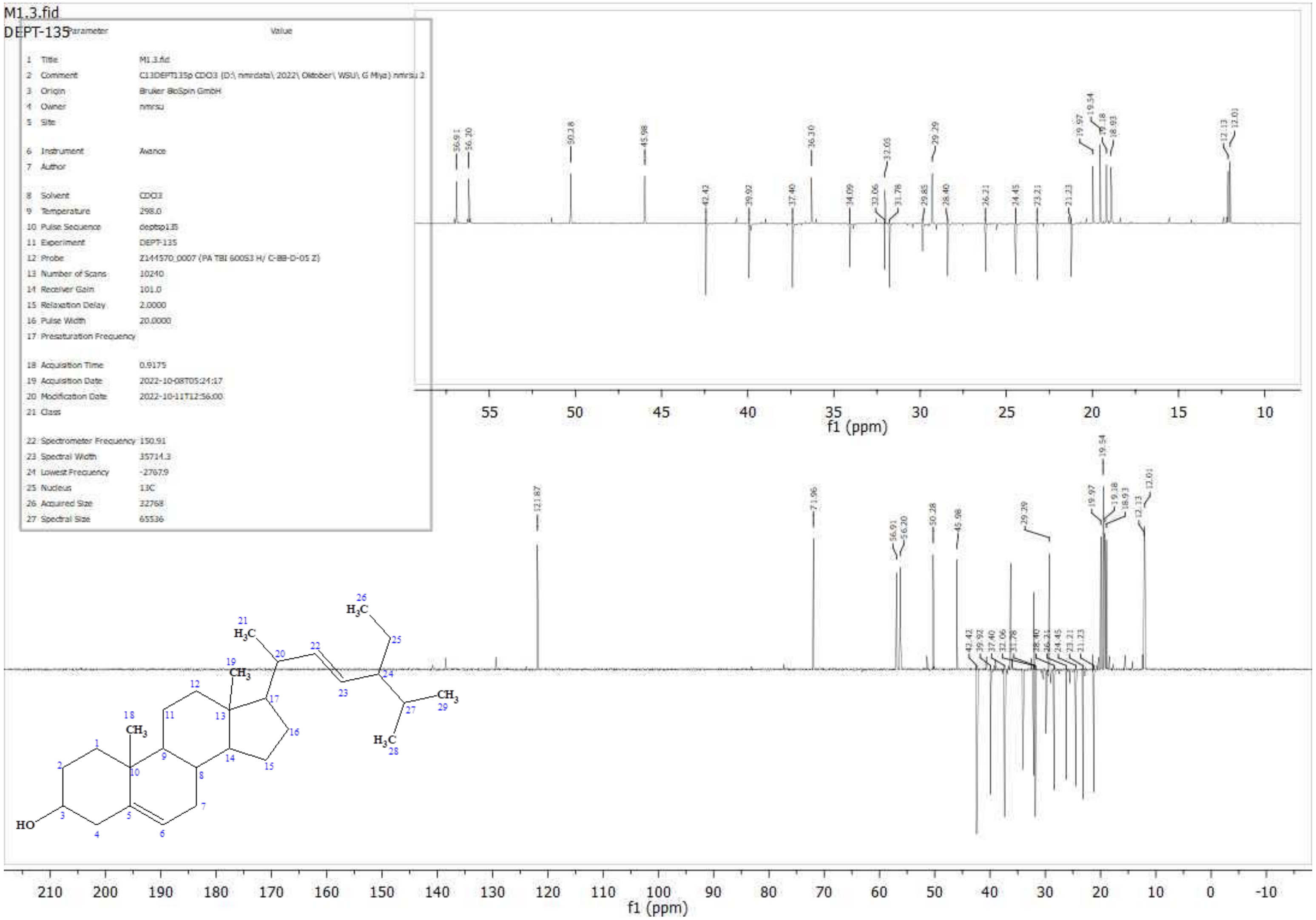

DEPT135 NMR Spectrum of Compound 1 – Stigmasterol from the n-Hexane Leaf Extract of CS

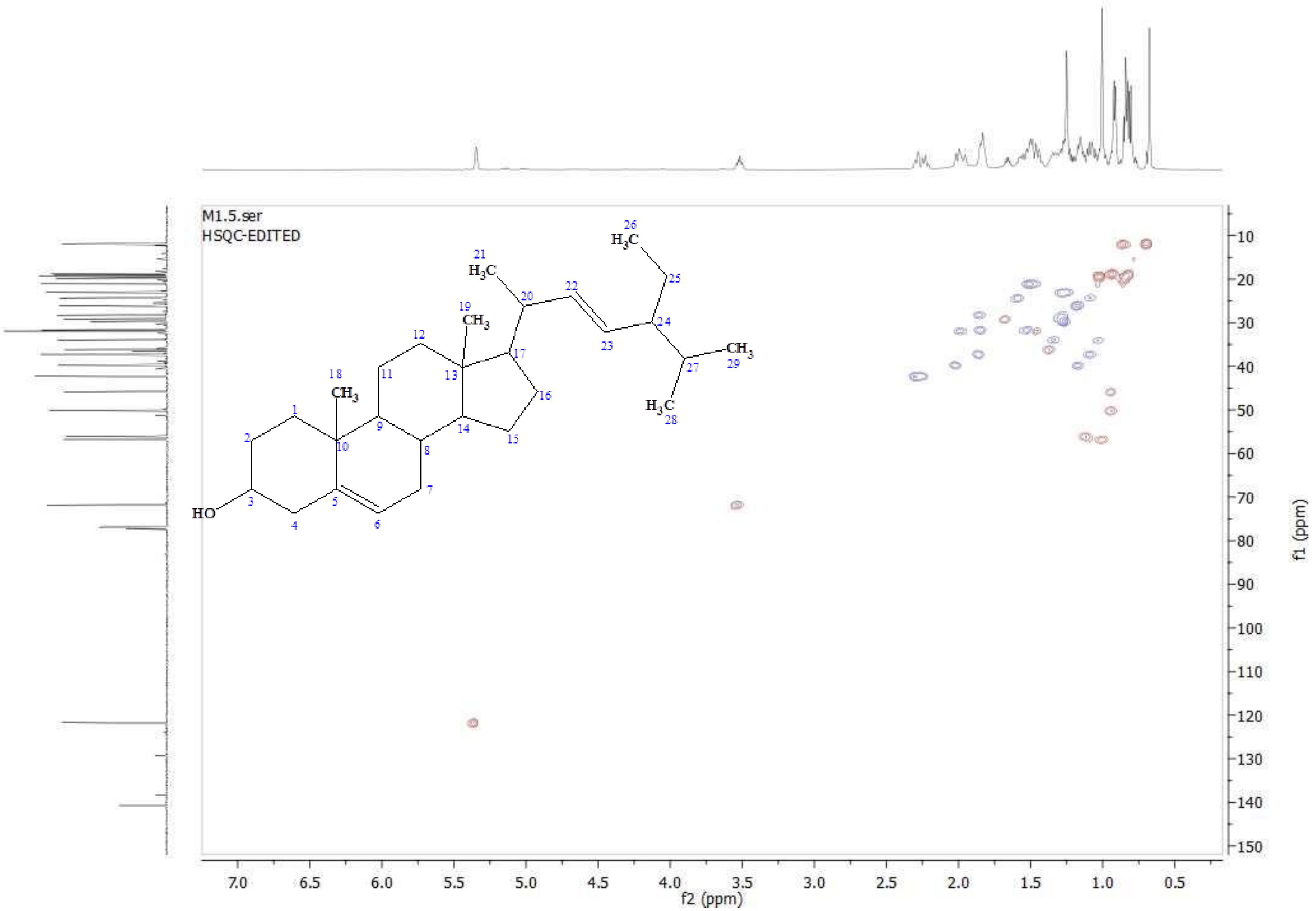

HSQC NMR Spectrum of Compound 1 – Stigmasterol from the n-Hexane Leaf Extract of CS

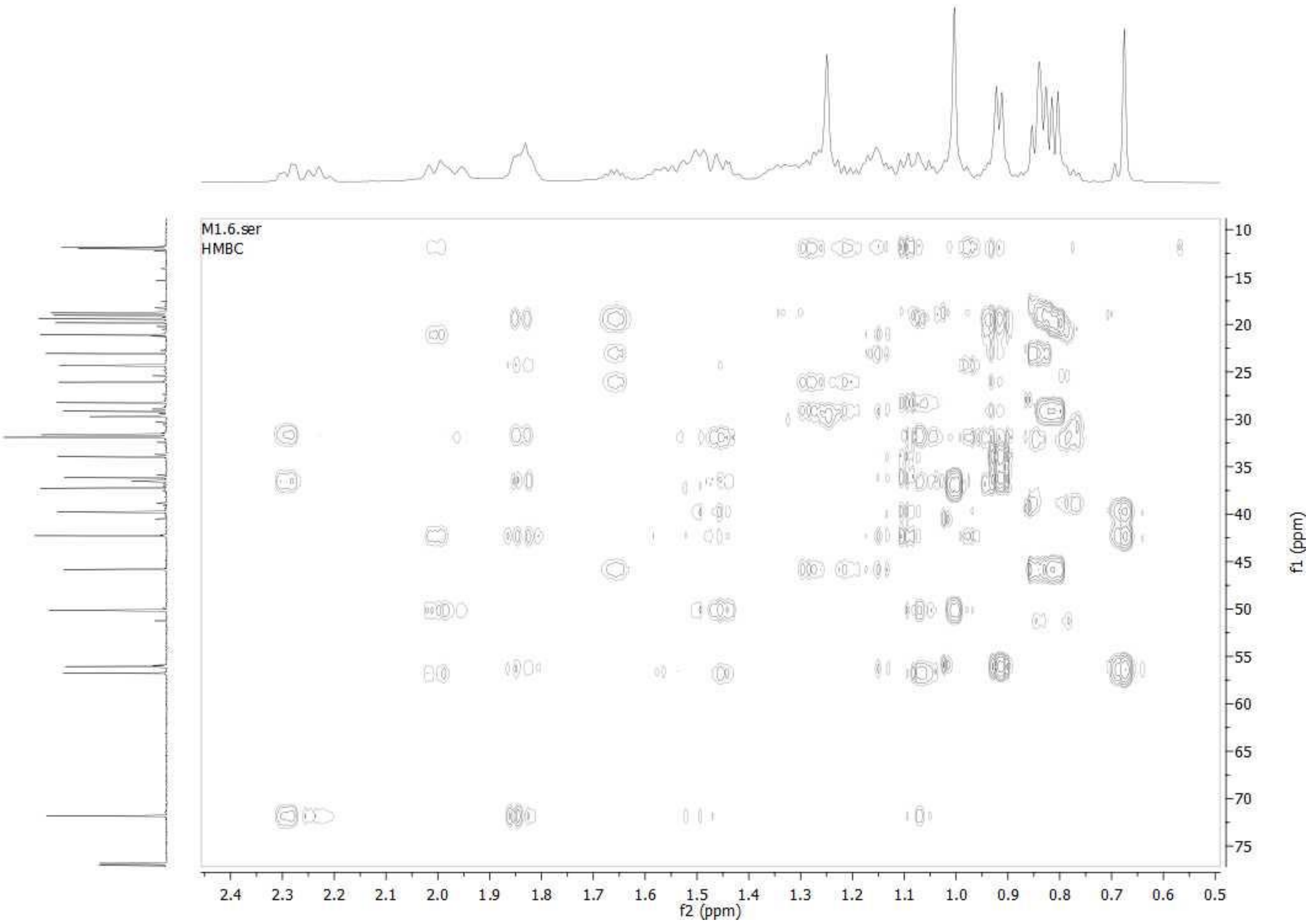

HMBC NMR Spectrum of Compound 1 – Stigmasterol from the n-Hexane Leaf Extract of CS

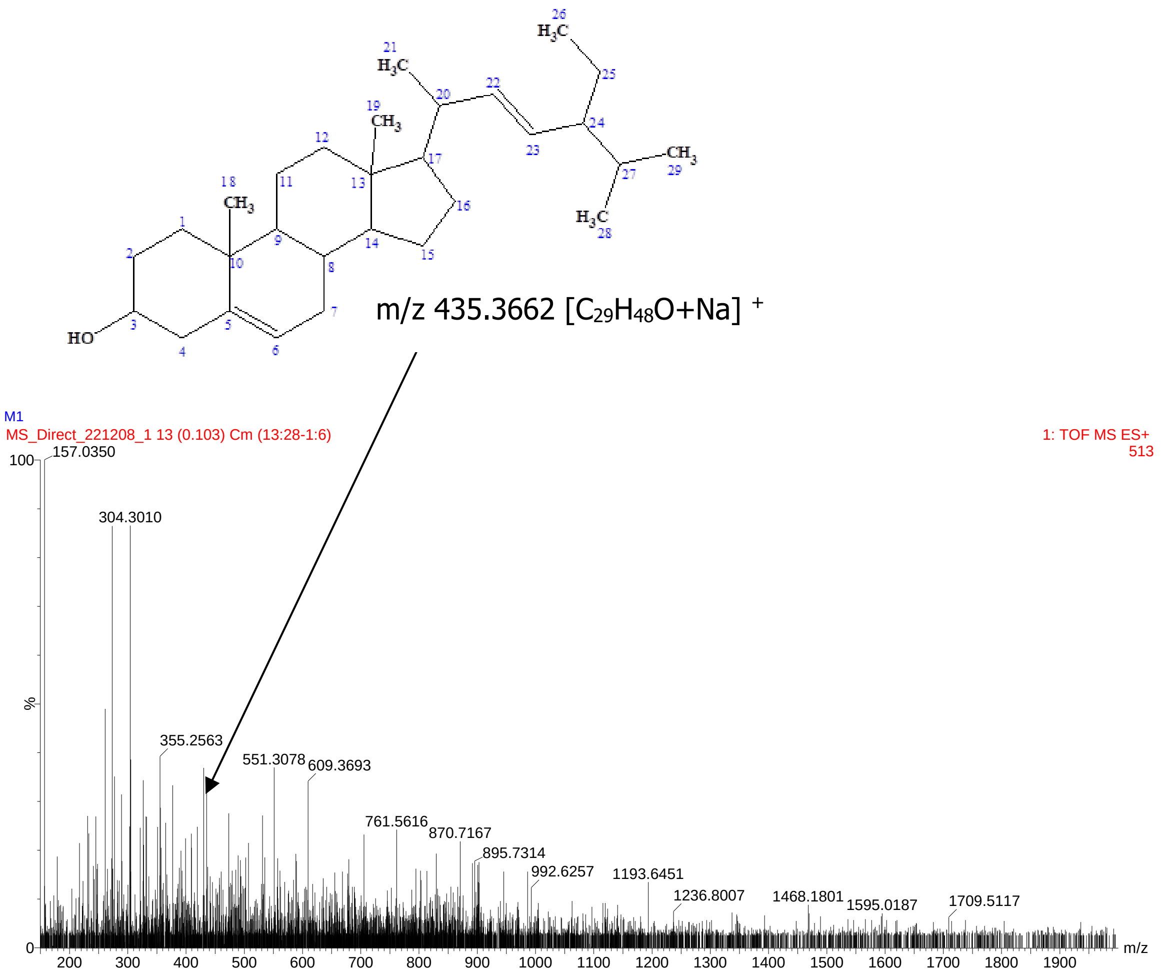

Mass [(TOF MS (ES+)] Spectrum of Compound 1 – Stigmasterol from the n-Hexane Leaf Extract of CS

Fig. S2

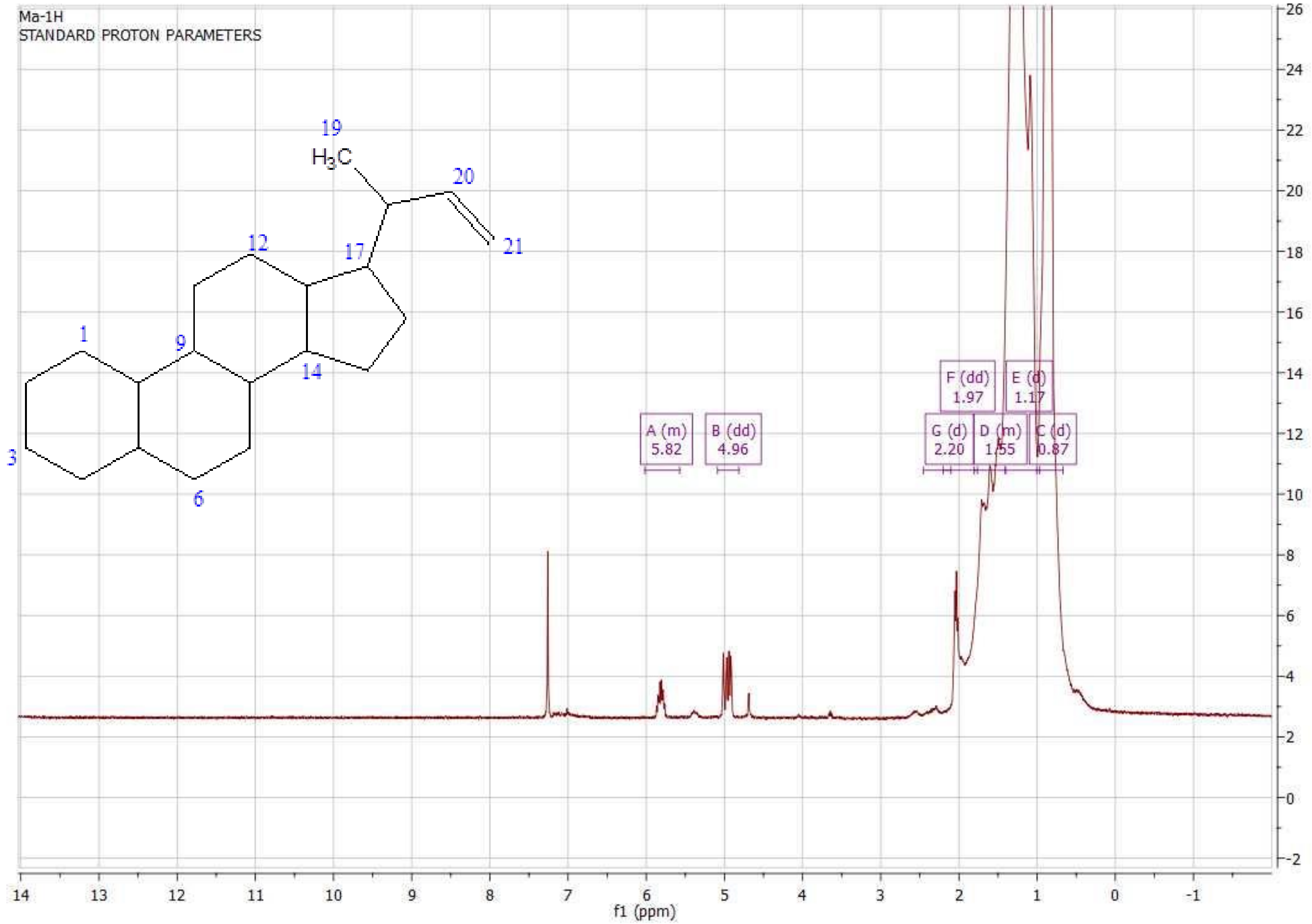

^{1}H NMR Spectrum of Compound 2– Phenanthrene Derivative from the DCM Leaf Extract of CS

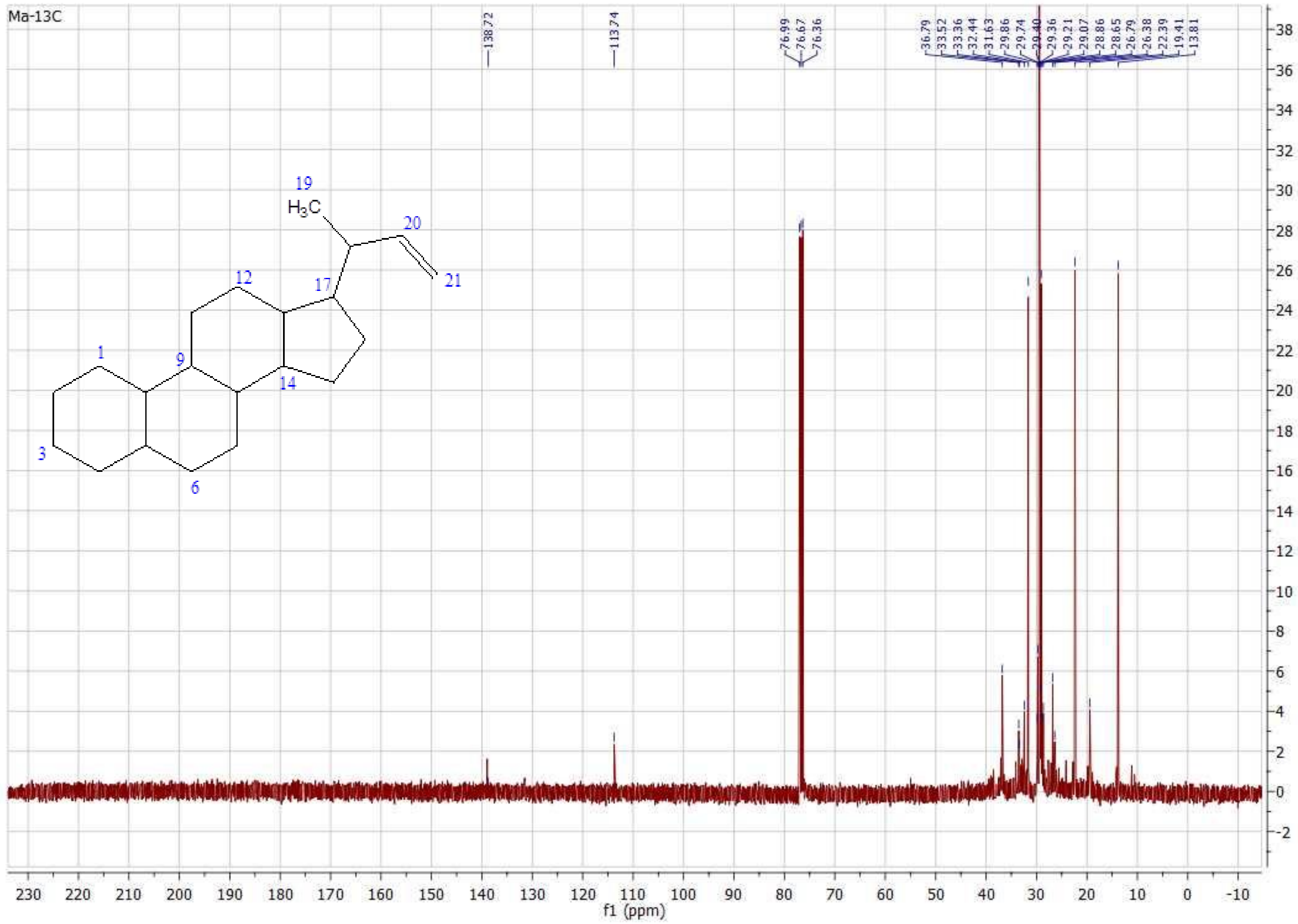

^{13}C NMR Spectrum of Compound 2 – Phenanthrene Derivative from the DCM Leaf Extract of CS

Fig. S3

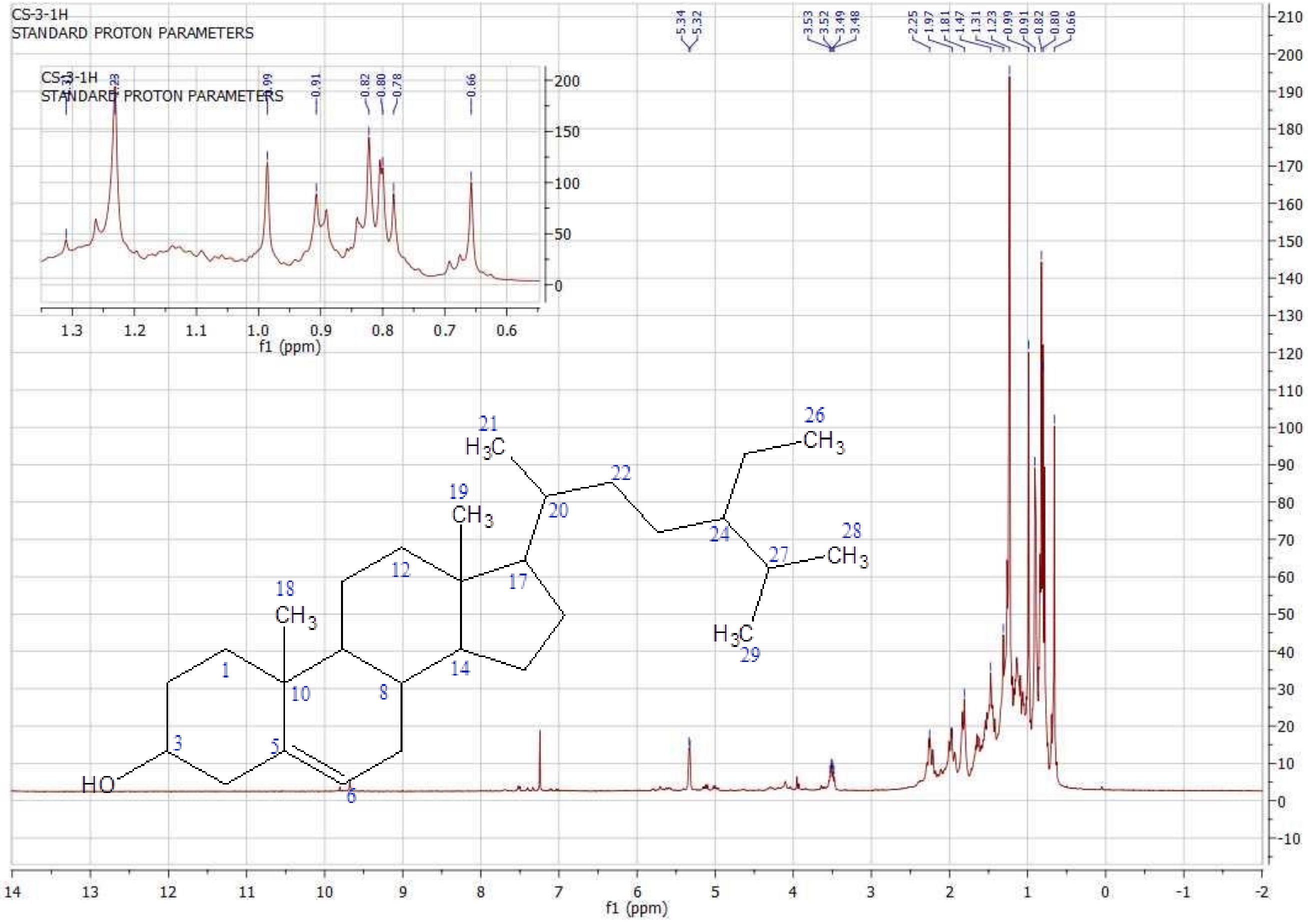

^{1}H NMR Spectrum of Compound 3 - β-Sitosterol from the DCM Extract of CS

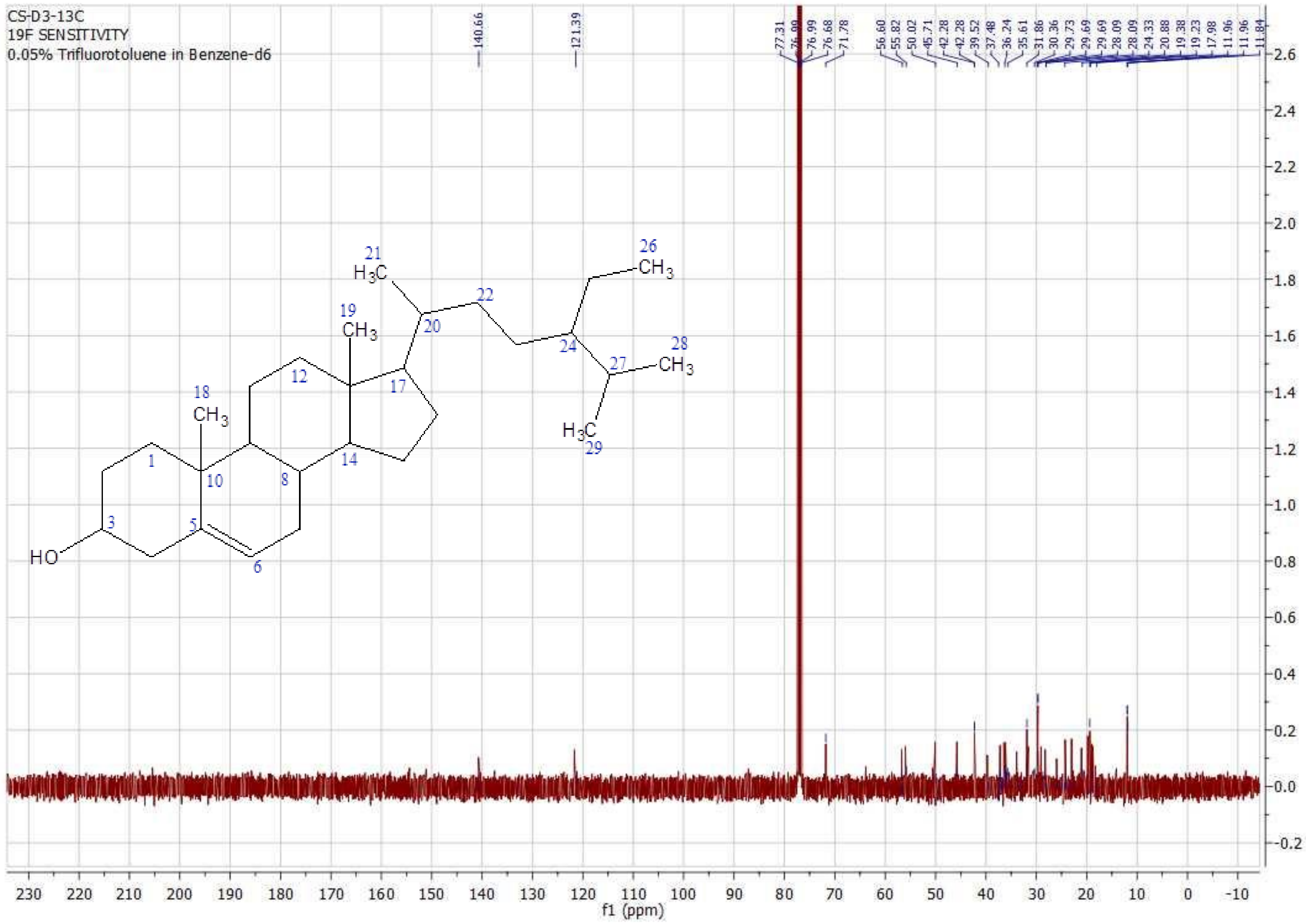

^{13}C NMR Spectrum of Compound 3 - β-Sitosterol from the DCM Extract of CS

Fig. S4

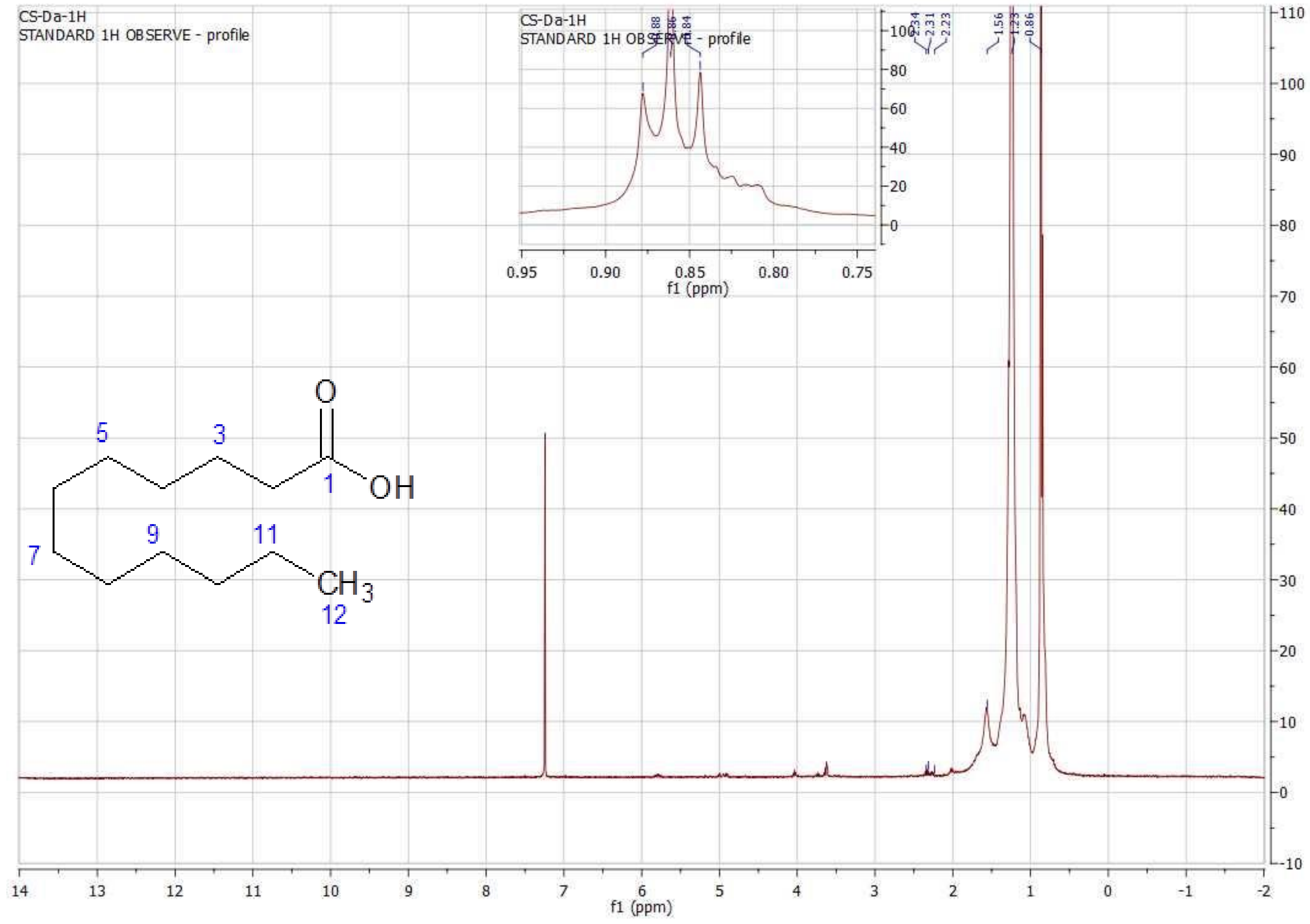

^{1}H NMR Spectrum of Compound 4 – Dodecanoic acid from the DCM Extract of CS

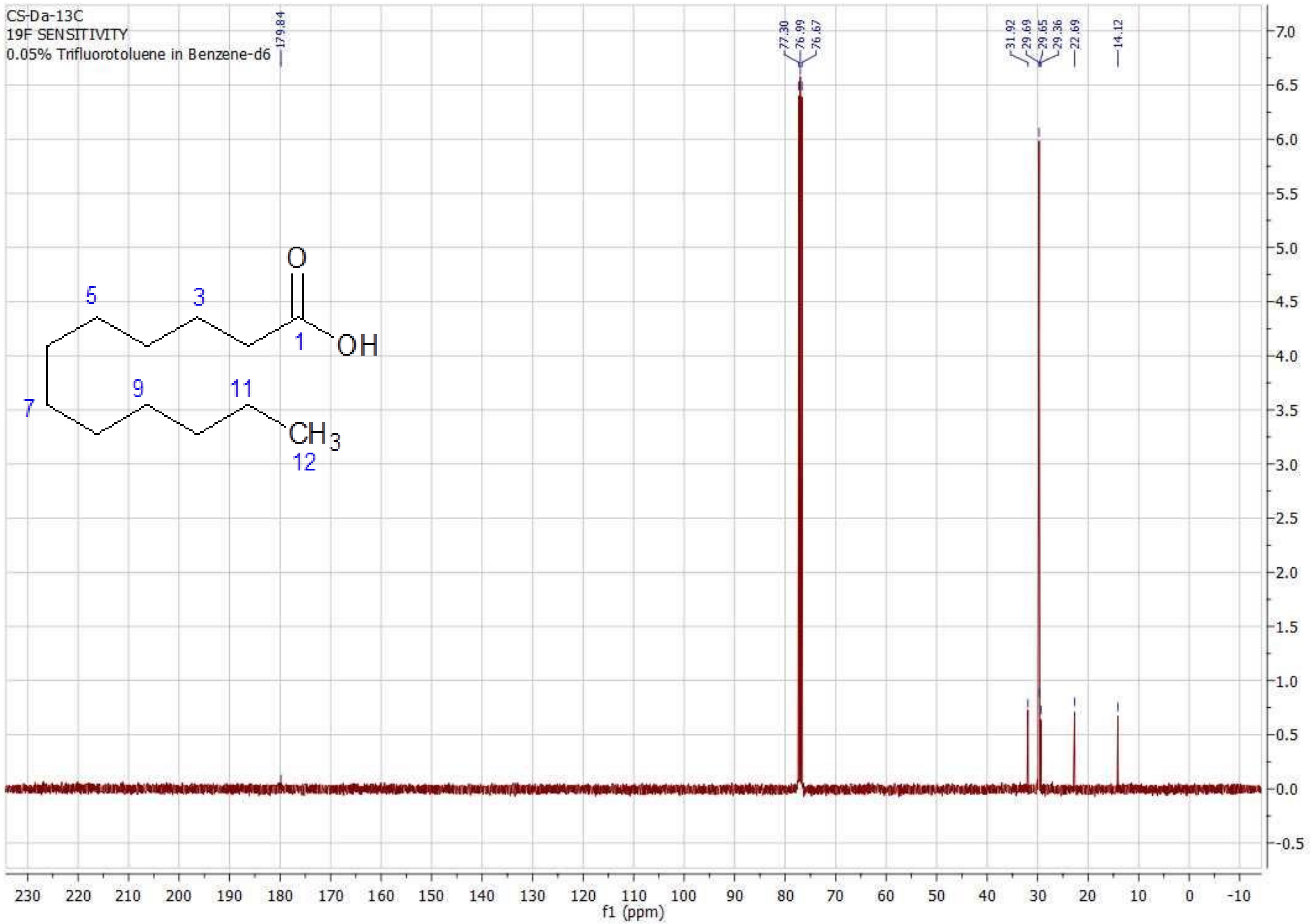

^{13}C NMR Spectrum of Compound 4 – Dodecanoic acid from the DCM Extract of CS

Fig. S5

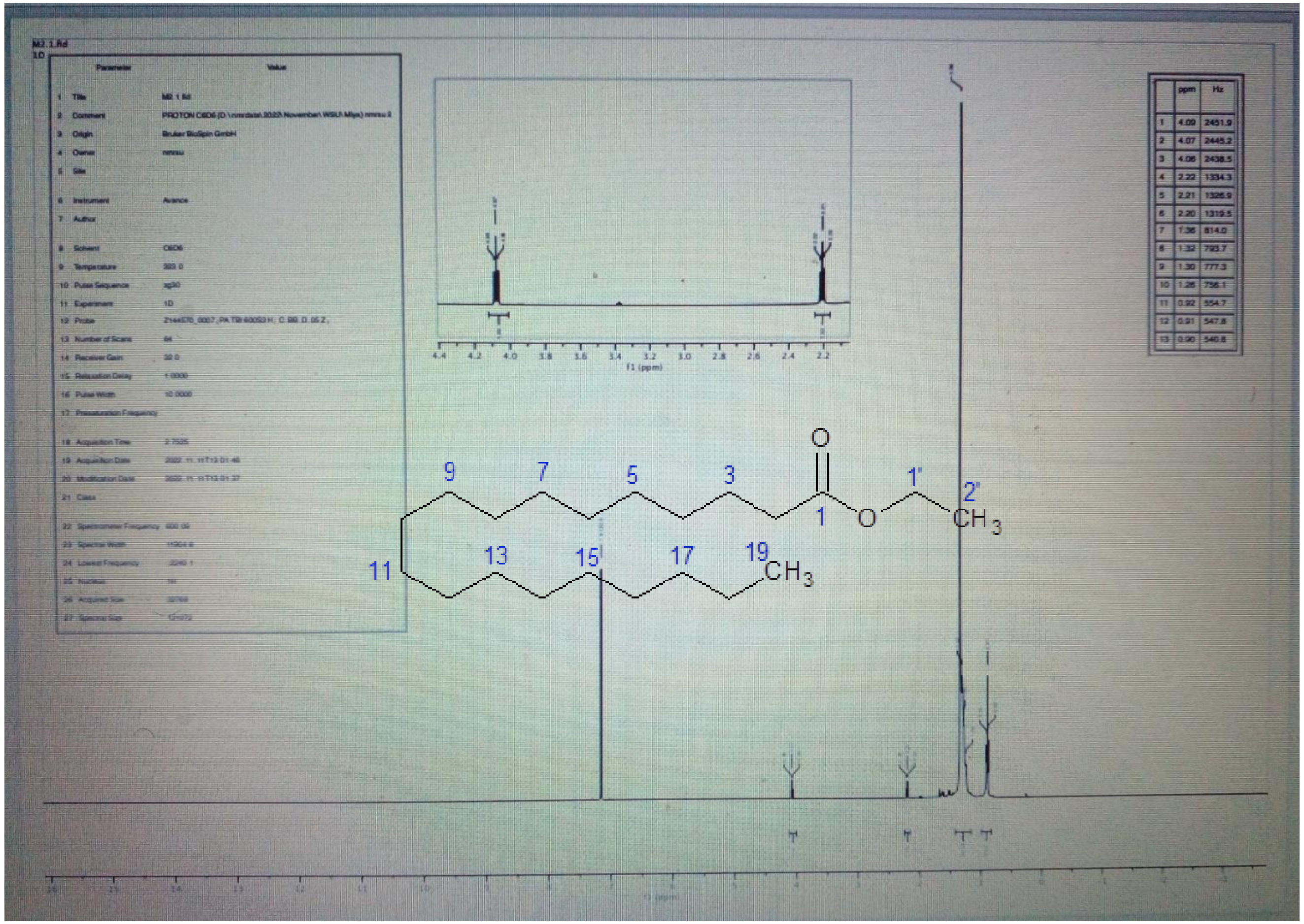

^{1}H NMR Spectrum of Compound 5 – Ethyl nonadecanoate from the DCM Leaf Extract of CS

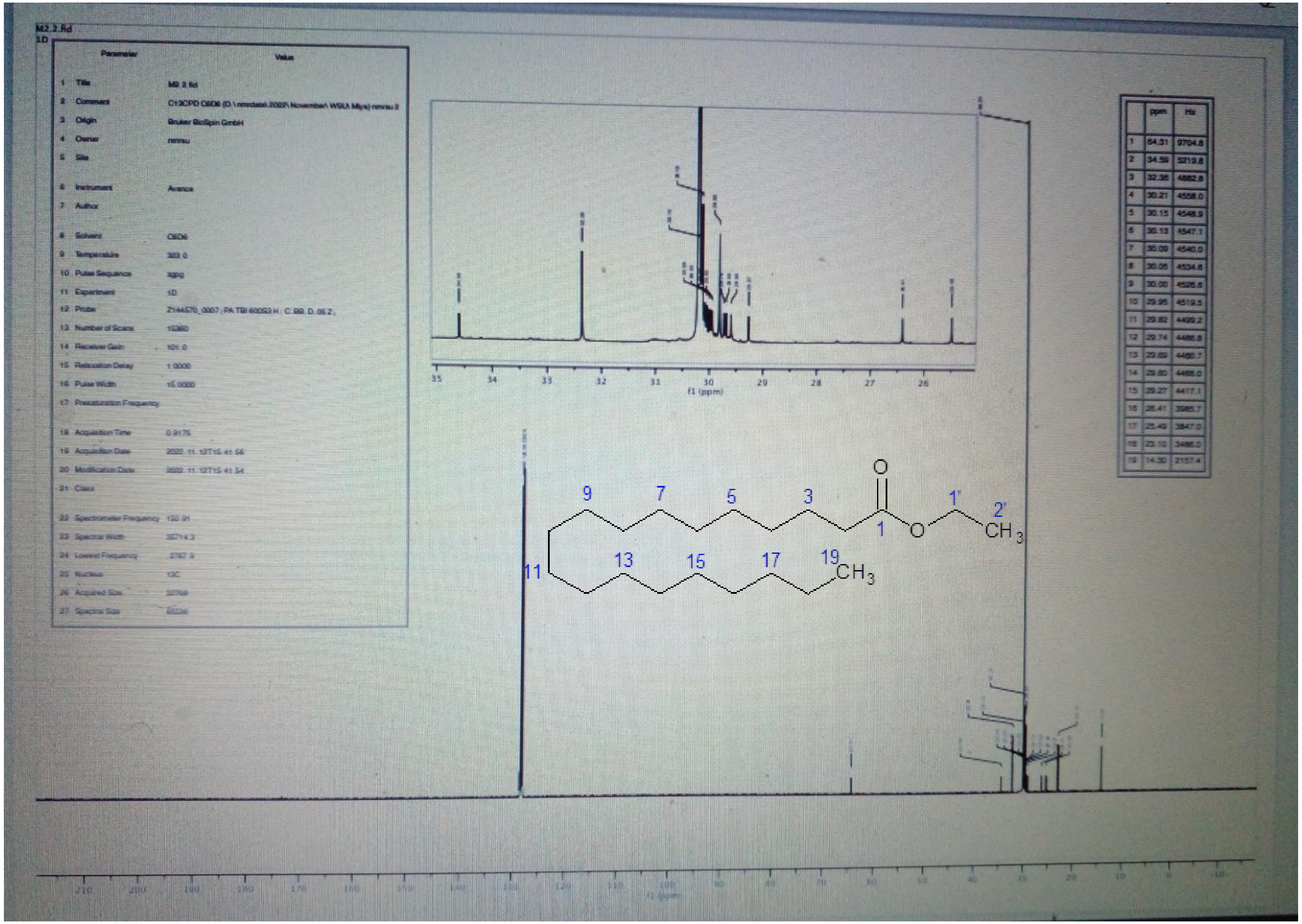

^{13}C NMR Spectrum of Compound 5 – Ethyl nonadecanoate from the DCM Leaf Extract of CS

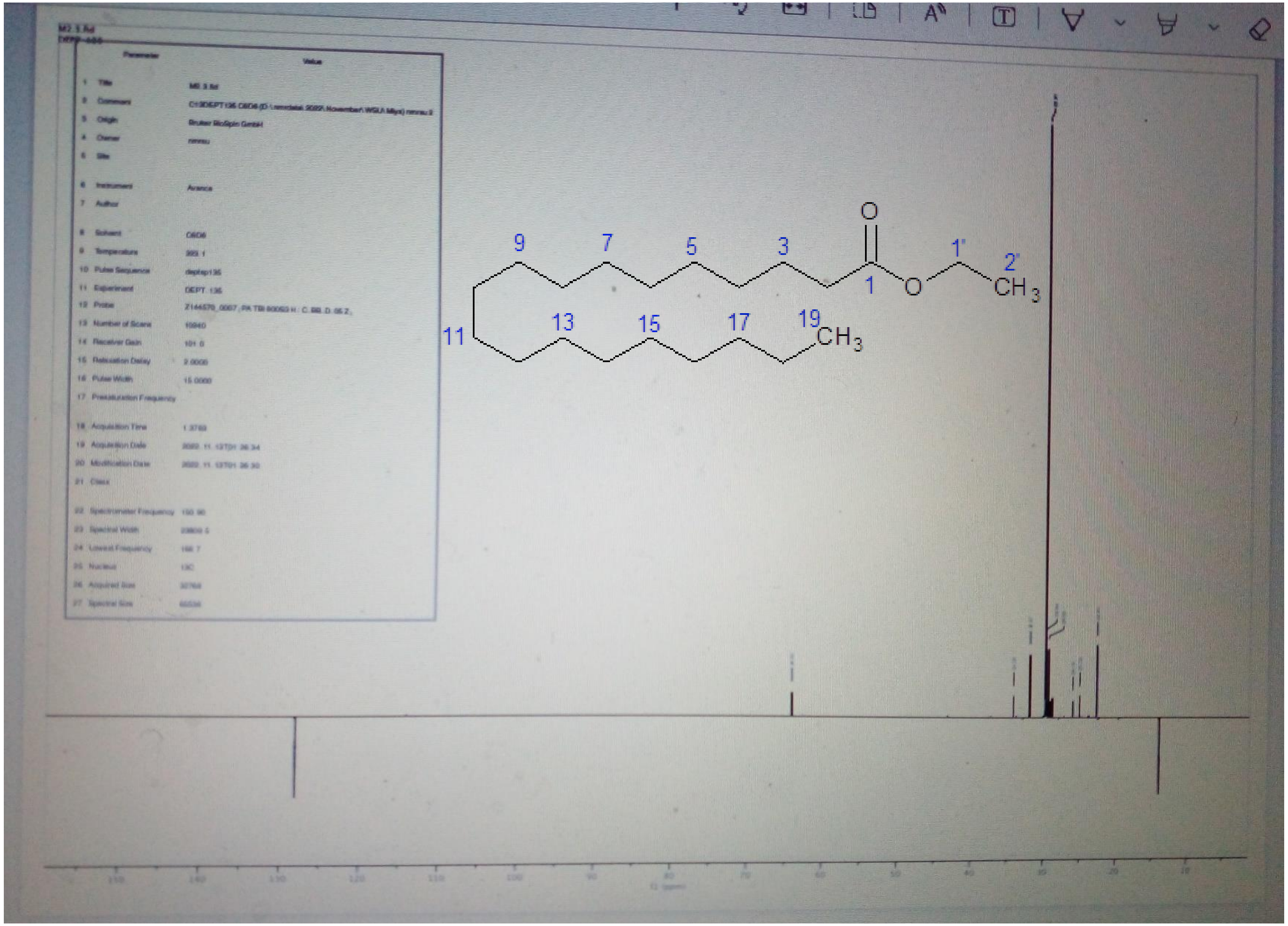

DEPT 135 NMR Spectrum of Compound 5 – Ethyl nonadecanoate from the DCM Leaf Extract of CS

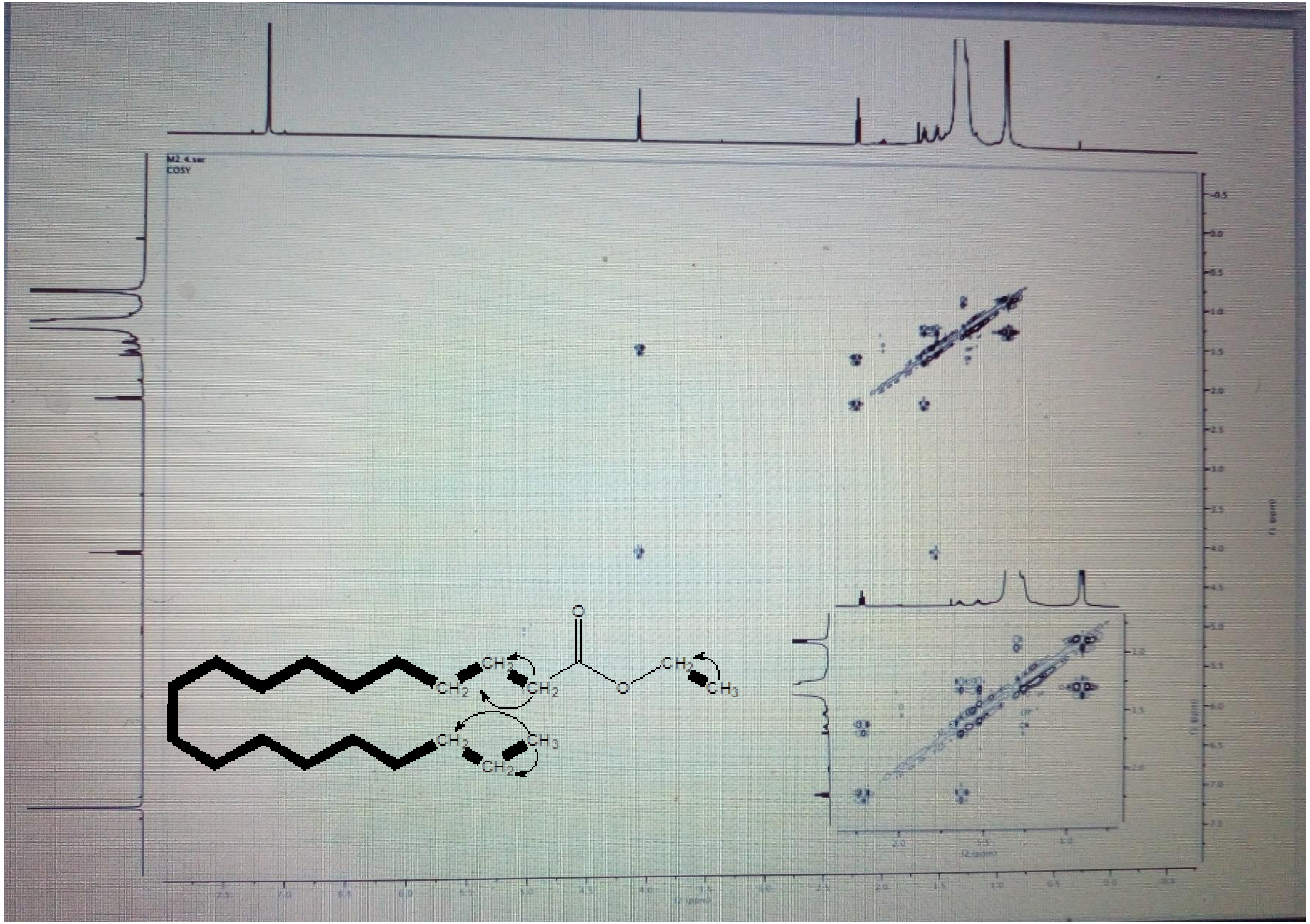

^1H ━ ^1H COSY NMR Spectrum of Compound 5 – Ethyl nonadecanoate from the DCM Leaf Extract of CS

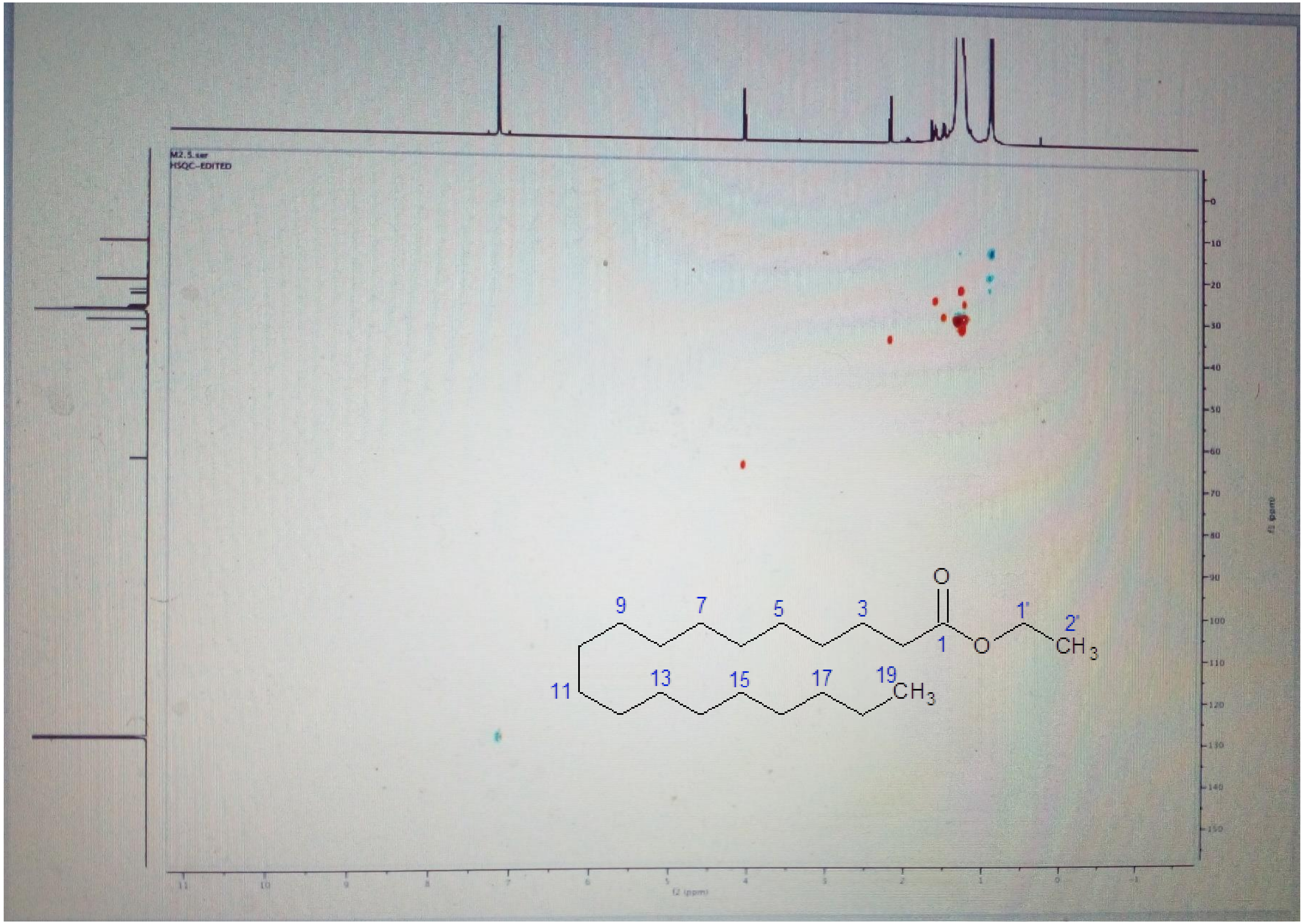

HSQC NMR Spectrum of Compound 5 – Ethyl nonadecanoate from the DCM Leaf Extract of CS

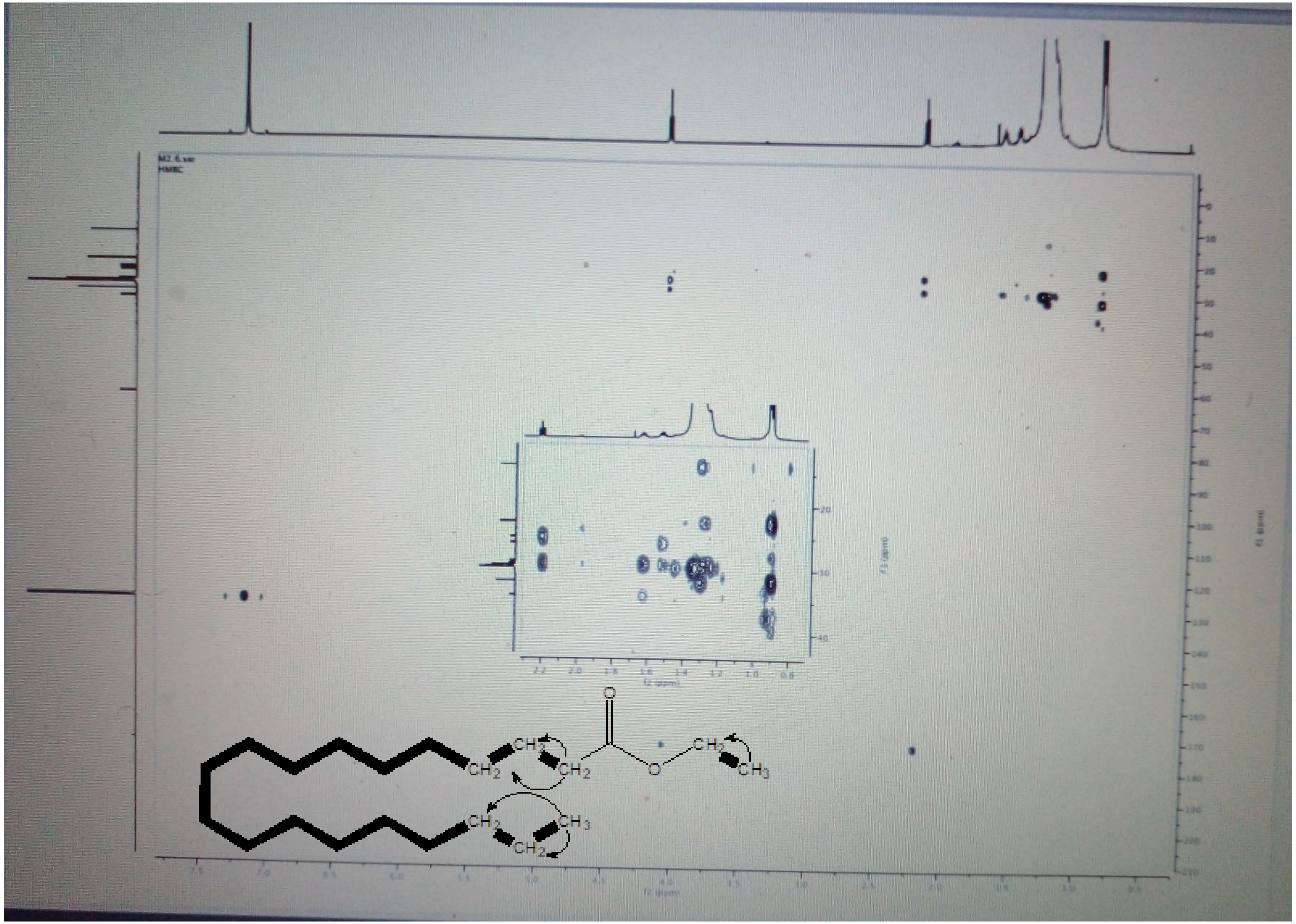

HMBC (C→H) NMR Spectrum of Compound 5 – Ethyl nonadecanoate from the DCM Leaf Extract of CS

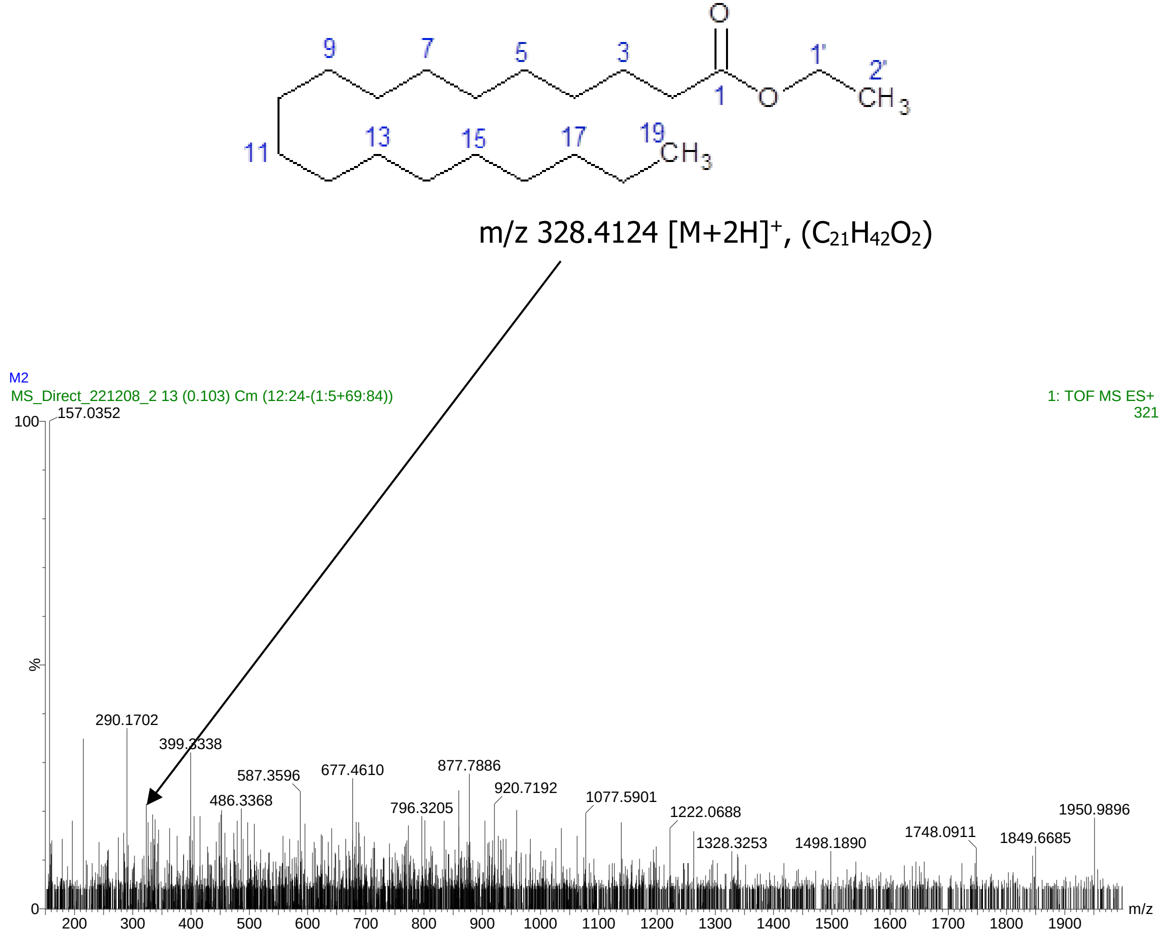

Mass [(TOF MS (ES+)] Spectrum of Compound 5 – Ethyl nonadecanoate from the DCM Leaf Extract of CS

Fig. S6

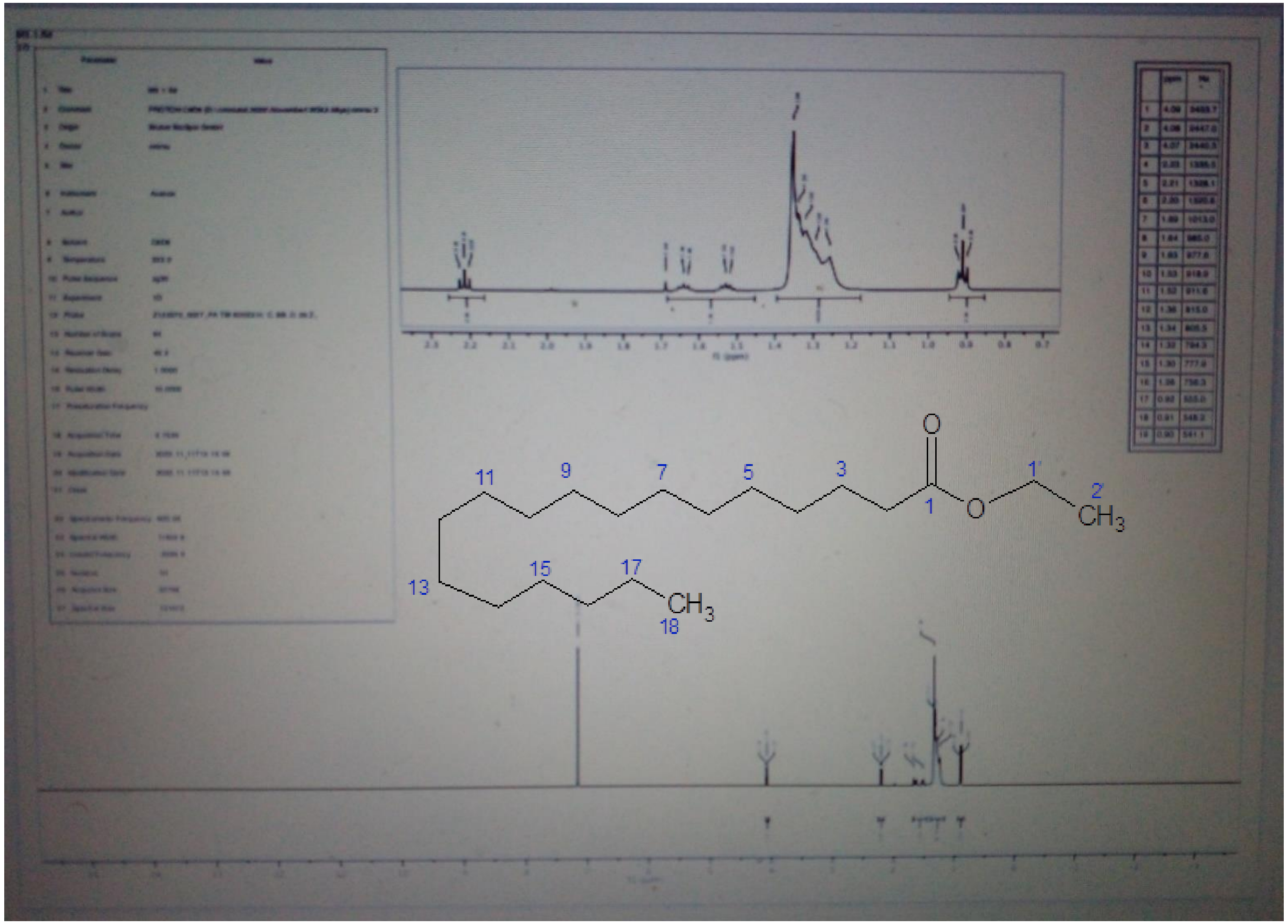

^{1}H NMR Spectrum of Compound 6 – Ethyl stearate from the DCM Leaf Extract of CS

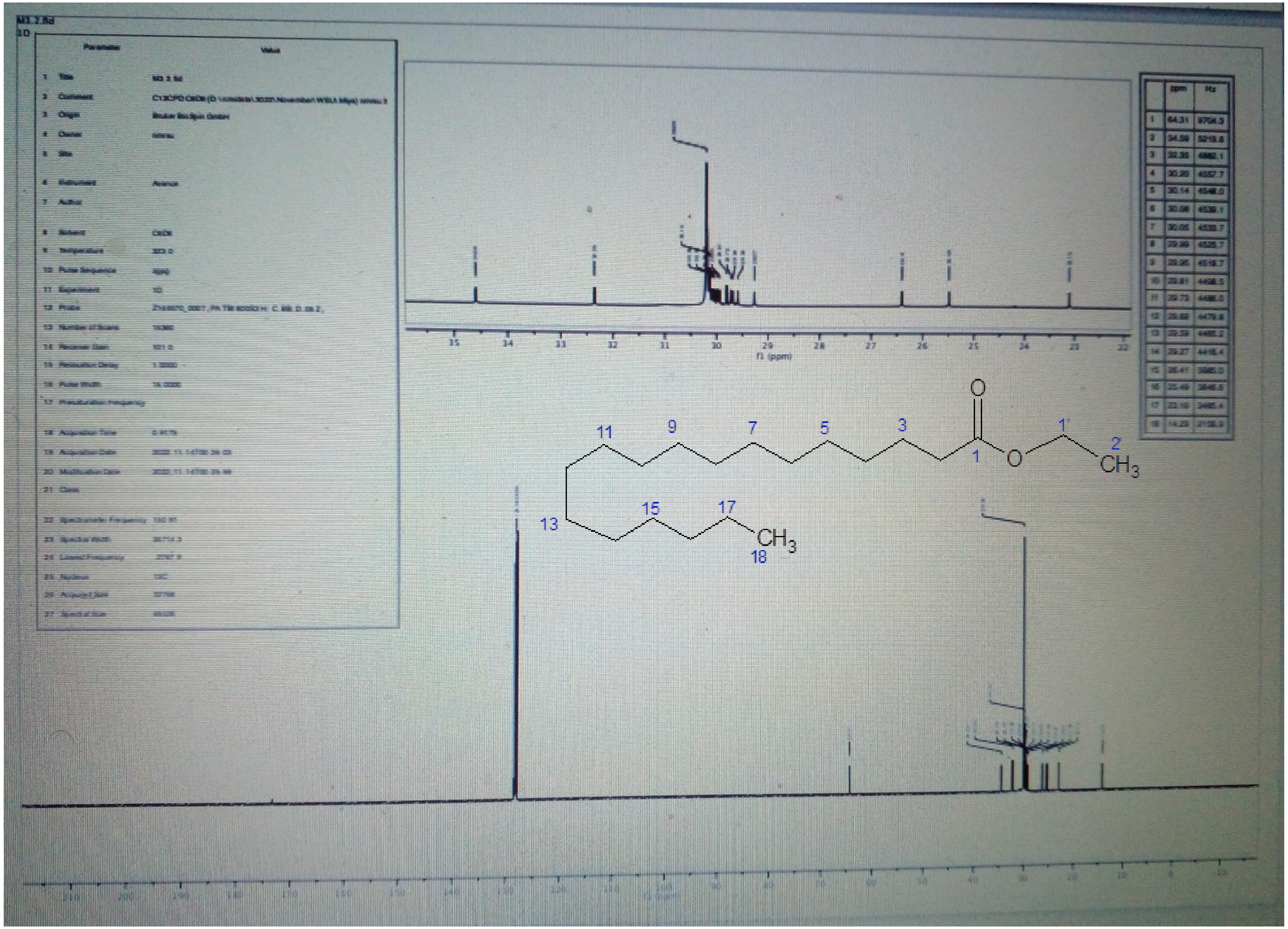

^{13}C NMR Spectrum of Compound 6 – Ethyl stearate from the DCM Leaf Extract of CS

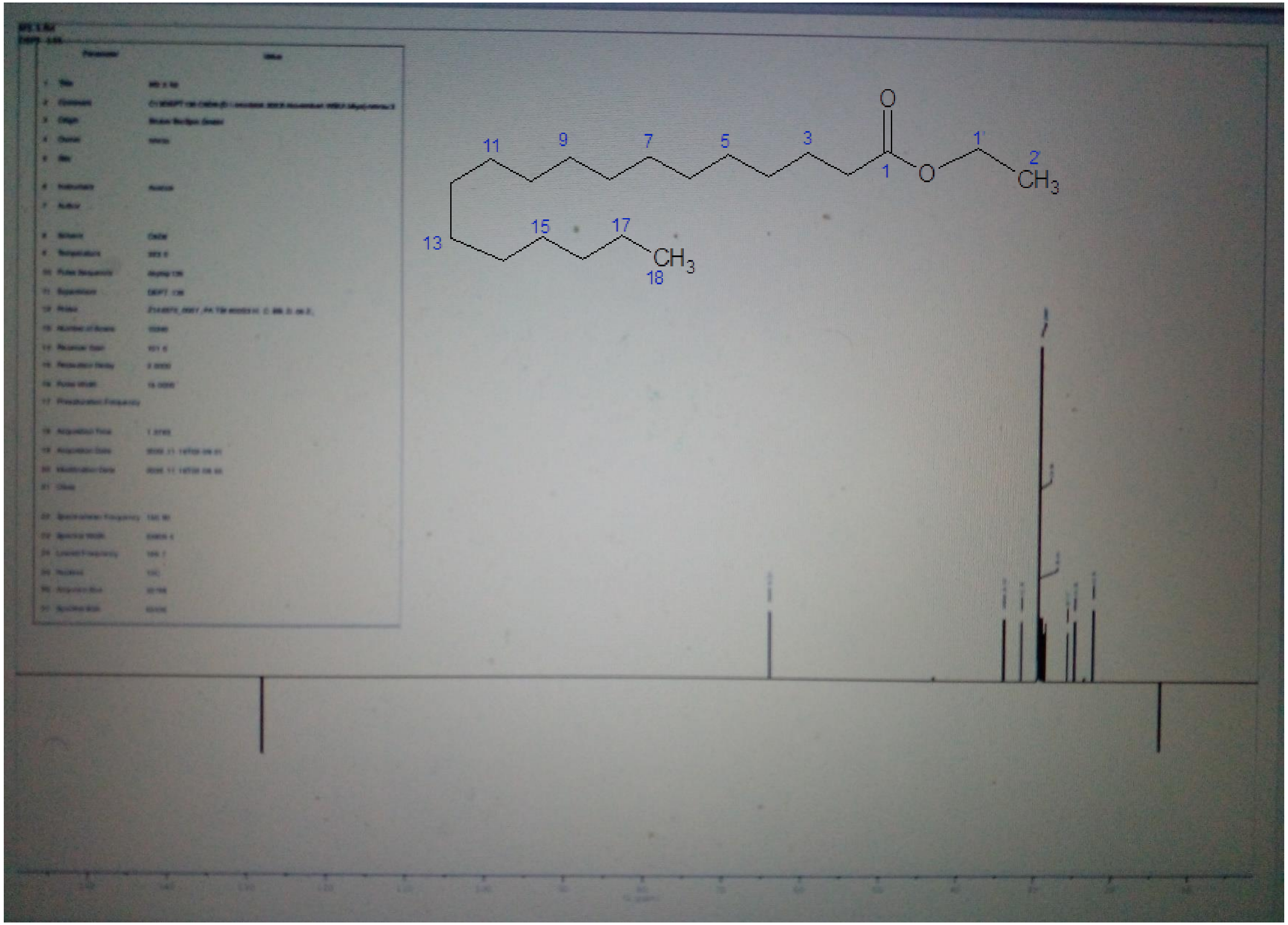

DEPT 135 NMR Spectrum of Compound 6 – Ethyl stearate from the DCM Leaf Extract of CS

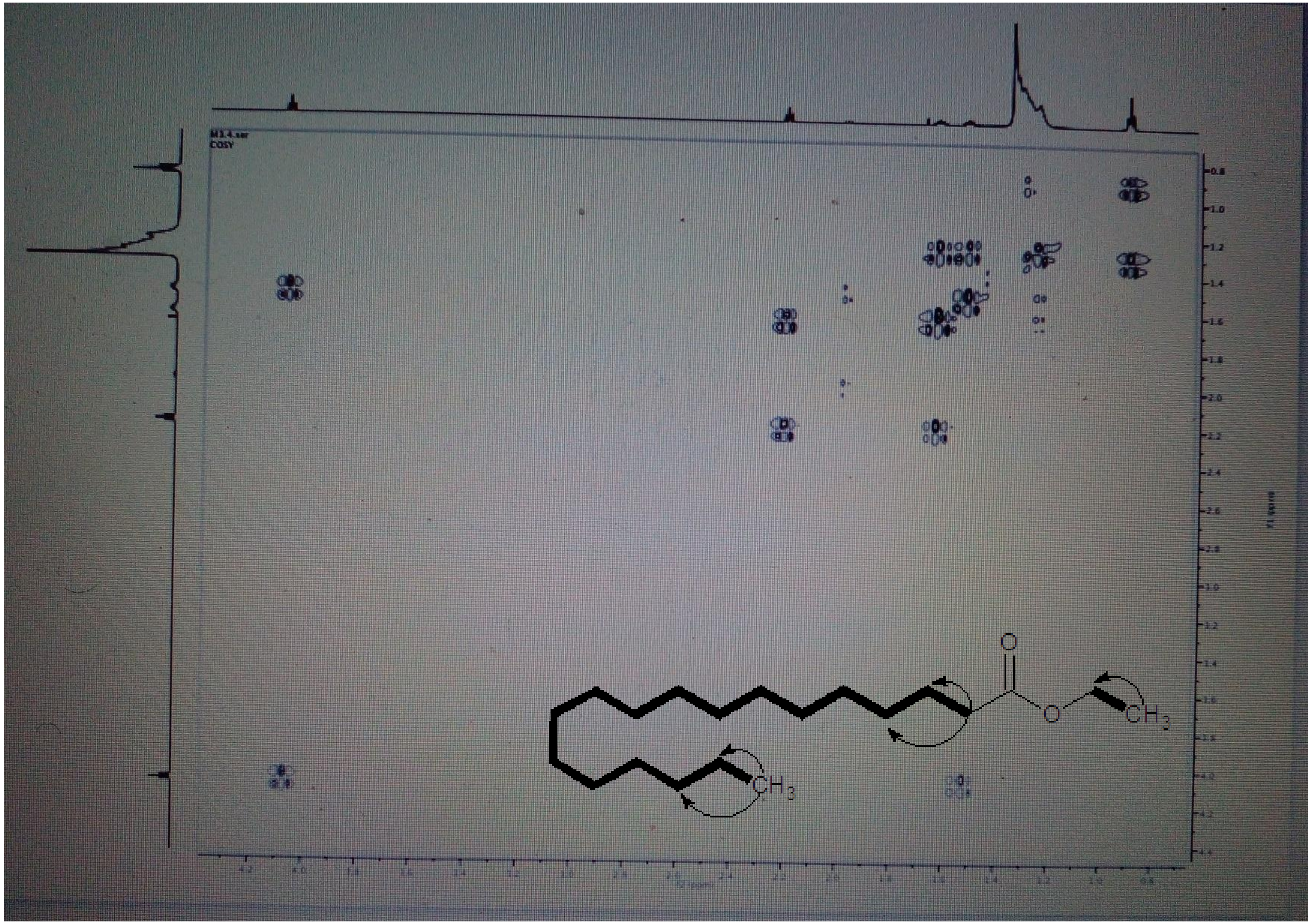

^{1}H — ^{1}H COSY NMR Spectrum of Compound 6 – Ethyl stearate from the DCM Leaf Extract of CS

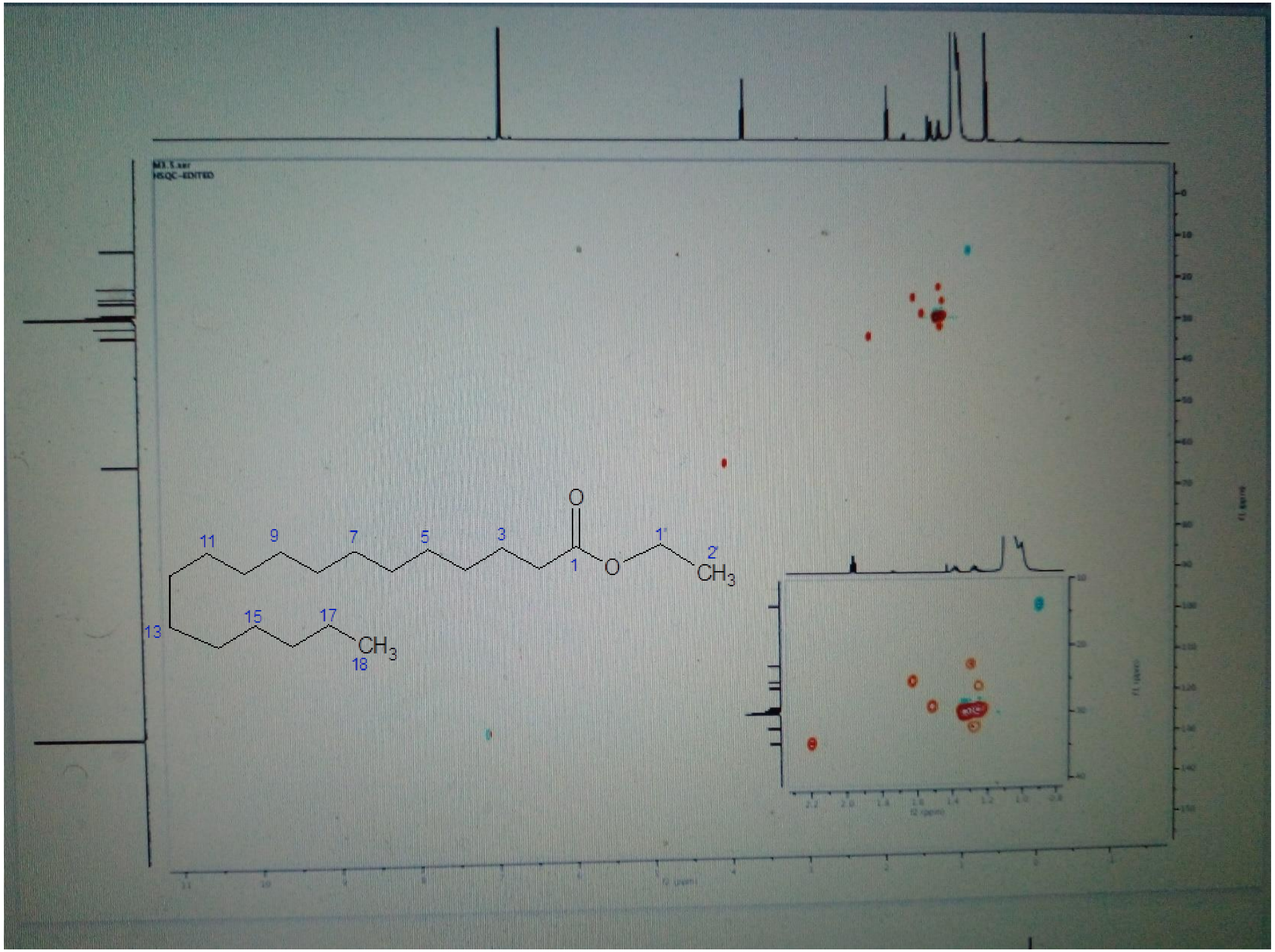

HSQC NMR Spectrum of Compound 6 – Ethyl stearate from the DCM Leaf Extract of CS

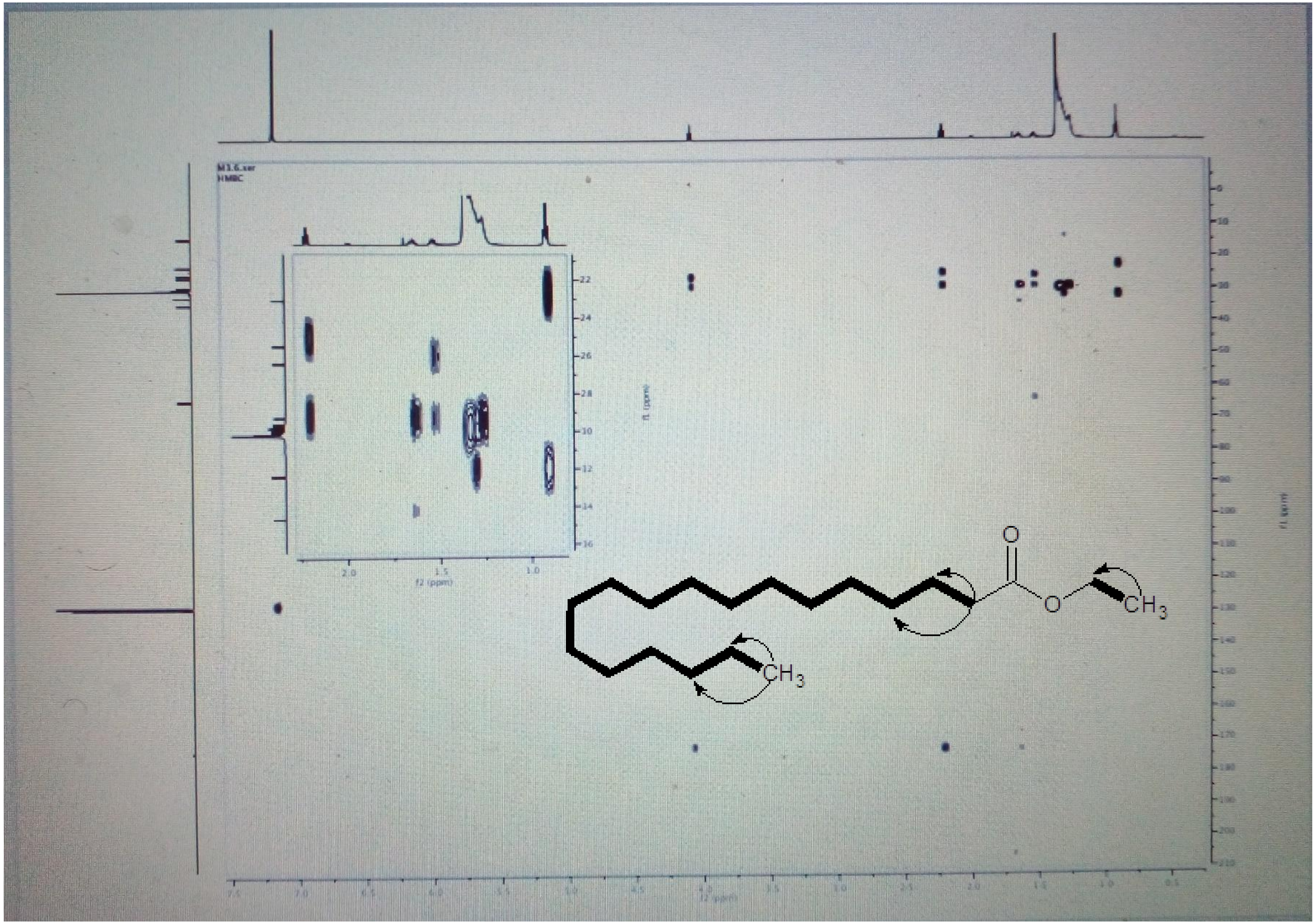

HMBC (C→H) NMR Spectrum of Compound 6 – Ethyl stearate from the DCM Leaf Extract of CS

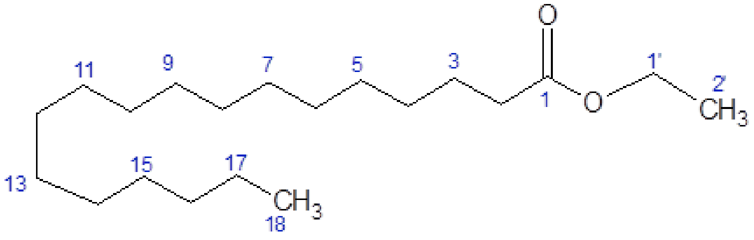

m/z 313.4124 $[M+H]^+$, ($C_{20}H_{40}O_2$)

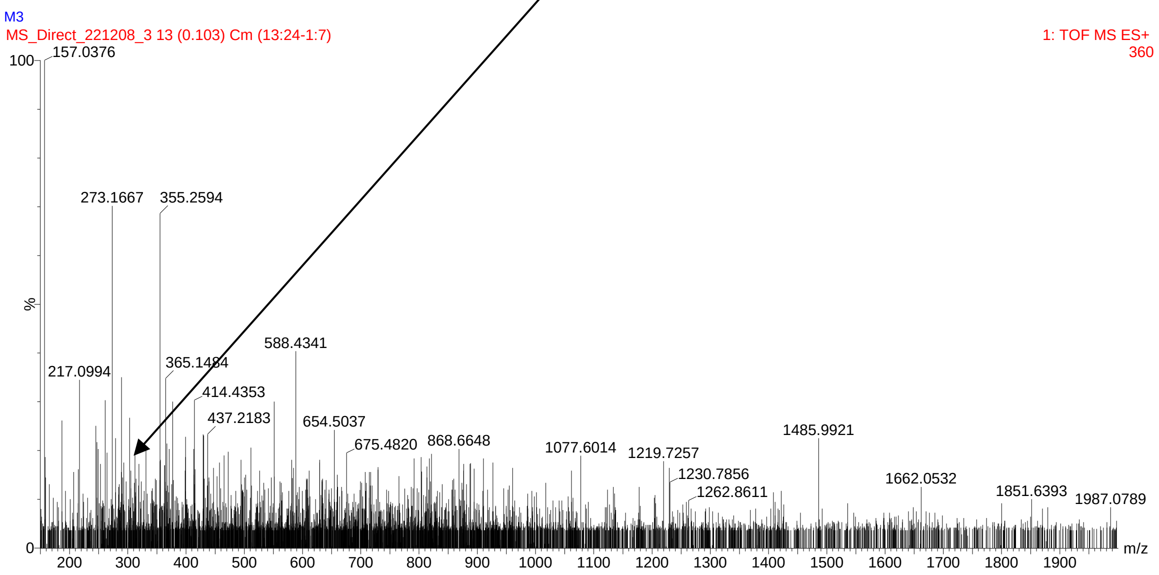

Mass [(TOF MS (ES+)] Spectrum of Compound 6 – Ethyl stearate from the DCM Leaf Extract of CS
